# Levels of Surgical Patients' Education Related to Surgical Interventions Among Patients in Saudi Arabia

**DOI:** 10.7759/cureus.42715

**Published:** 2023-07-31

**Authors:** Jubran Jaber AlFaifi, Rawan Abdulrahman Alrehaili, Shaden Dhafallah Alshammari, Jumanah Hamed Mohammed Alqurashi, Haifa Mohammed Ali Alasmari, Afnan Fahad Saleh Alhallafi

**Affiliations:** 1 Department of Surgery, College of Medicine, Imam Mohammad Ibn Saud Islamic University, Riyadh, SAU; 2 College of Medicine, Imam Mohammad Ibn Saud Islamic University, Riyadh, SAU

**Keywords:** surgery, saudi arabia, patient, intervention, education

## Abstract

Background: Patient education and counseling should simplify and clarify the condition, surgery, postoperative care, and potential complications. This study aimed to determine the levels of surgical patients' education regarding surgical interventions among patients in Saudi Arabia.

Method: This was an online survey study that was conducted between January and May 2023 to determine the levels of surgical patients' education regarding surgical interventions among patients in Saudi Arabia. The study population was patients who underwent surgeries (elective and emergency) living in Saudi Arabia. The questionnaire tool used in this study was developed based on an extensive literature review in the field of patients' education regarding surgical interventions. Binary logistic regression analysis was used to identify predictors of satisfaction with the callouts and the surgeon-consultant's communication quality.

Results: A total of 1360 participants were involved in this study. Around 40.5% of the participants reported that they met the surgeon after diagnosing and recommending the issue. Almost 70.0% of research participants reported that the consultant surgeon personally explained a diagnosis or strategy to attain it and the surgical technique, method, and purpose before signing the informed consent. The majority of study participants reported that the consulting surgeon or a member of his surgical team explained the stages of reaching a diagnosis and the steps he/she will take to diagnose their condition (83.2%), discussed the diagnosis with them and how certain they are of the diagnosis (88.1%), described the surgery clearly and simply (85.5%), informed them of alternatives to surgical intervention (63.1%), and discussed the entire postoperative treatment plan (81.8%), informed them of possible postoperative complications (79.6%), used additional callout during the discussion (81.3%), and spoke to them after the surgery and before they left the hospital (69.2%). After a conversation with their doctors, 36.3% of study participants said they did not require an outside source to understand the diagnosis, operation, probable problems, treatment plan, and follow-up. Discussion "Just talk," sketching, and pictograms were the most popular callouts during patient education, with 78.3%, 22.3%, and 17.9%, respectively. Saudi participants were more likely to be satisfied with the quality of communication by the surgeon-consultant (p<0.05). At the same time, participants who live in the Southern area were less likely to be satisfied with the quality of communication by the surgeon-consultant (p<0.05).

Conclusion: This study highlights the crucial role of surgeons in preoperative patient education as well as the significance of surgical team participation in this process. In order to increase patient knowledge, facilitate treatment decisions, and assure informed consent, it is necessary to establish guidelines and roles to improve surgeon-patient communication, increase patient and surgeon awareness, nurture patient concern expression, and encourage non-medical patient participation.

## Introduction

To enhance the outcomes of healthcare, physicians must spend more time with patients. The interaction between the teaching physician and the patient must be enthusiastic, motivated, and sensitive to the patient's requirements. Physicians must promote patient education and engagement by improving the health literacy of their patients. Health literacy is the capacity to locate, comprehend, and utilize health information [1]. A correctly informed consent procedure must begin with patient education prior to any medical intervention. In addition, through education and counseling, a comprehensive understanding of the patient's condition should be developed, encompassing every aspect of care. In addition, there are numerous options for formal and informal education and counseling, including personal verbal interaction between the patient and the healthcare professional, detailed discourse facilitated by hand drawing, external illustration, educational medical models, publications in the form of booklets, brochures, or pamphlets, digital forms, such as custom-made films and multimedia applications, or a combination of other techniques. In addition, surgical intervention interacts with the informed consent procedure multiple times per day [2]. Cholecystectomy is a very common elective surgical intervention used to treat cholelithiasis (gallbladder stone). Prior to the informed consent process, the patient should be given a clear explanation of the condition, the surgery, postoperative care, possible outcomes, and complications [3,4]. In patient education, identifying the source of information is equally as important as the method employed [5].

Numerous investigations have been performed. Prior to undergoing laparoscopic cholecystectomy, a study of 100 patients was conducted in 2006 to identify the sources and content of health information obtained by patients. The majority of respondents cited their physicians as their source of information. Around 21% consulted additional resources, such as the laparoscopic cholecystectomy information leaflet, the internet, and medical literature. In a few instances, medical students and the admissions clerk served as additional sources. In addition, when patients were asked about their preferable source of information, 35% said health professionals, 33% said printed literature, and 13% said videos [6].

After employing a wide range of preoperative education techniques to establish the informed consent procedure, a number of studies have been conducted to evaluate the patients' knowledge using questionnaires that were developed specifically for this purpose. A study in which an interactive computer program was used to educate 257 patients found that a large number of patients rated the information, text, and illustration clarity and suitability to their prior knowledge as good or excellent and would prefer to be educated again using the same method [7]. In addition, 60 participants undergoing elective laparoscopic cholecystectomy were randomized into four groups: A, B, C, and D. Group A was informed by a multimedia health education program, Group B was given a leaflet containing the precise contents of the multimedia health education program, Group C was informed verbally by a registered nurse, and Group D, the control group, was informed only by the attending surgeon. The questionnaire revealed that the information provided by the attending surgeon was insufficient in comparison to the specifically developed multimedia health educational program, which also appears to be effective in reducing preoperative anxiety and postoperative pain in patients [8,9].

A similar study involving 40 patients prior to gastric banding evaluated a multimedia program based on the lucidity of the information as well as their level of satisfaction and apprehension during the informed consent procedure. Twenty of these patients were provided with conventional documents, while the remaining twenty were provided with a multimedia program that covered the preoperative examination, the procedure itself, hospital stays, surgical risks, alternative treatments, pathophysiology, and health risks of obesity. The results indicated that there was no discernible difference between the apprehension levels of the two groups [10]. However, the group informed by the multimedia method demonstrated a significantly higher level of contentment with the informed consent procedure and a greater comprehension of the material.

The study indicated that the use of multimedia-based programs has a significant impact and enhances patients' knowledge of the disease and its treatment, particularly those with less formal education. Nonetheless, there was no difference in apprehension levels [11]. Again, a randomized controlled trial involving 238 patients examined the use of multimedia DVD education on 140 of them and then assessed the patients using a special questionnaire. The outcome had a positive effect because it increased the patients' knowledge and was simple to implement. In spite of this, multimedia tools should be used to support or aid the consent process and not as a replacement for personal interaction, as engaging with the physician is beneficial for patients because it enables them to ask questions or clarify unclear thoughts, misunderstandings, or beliefs [12].

A 2011 review of communication methods sought to enhance patient comprehension during the informed consent procedure for medical and surgical interventions. Among the included methods are written information, digital media such as audio-visual or multimedia, extended discussions, testing, and feedback [13]. The review concludes that a variety of formal and informal communication techniques improve patients' understanding of the informed consent procedure. Schenker et al. suggested that, in order to enhance the informed consent procedure, various aspects of method comprehension, probability, and acceptability should be considered [14]. In addition, the purpose of the study was to determine how patients understand the informed consent procedure and how it affects their decision to undertake elective surgery. Using a telephone interview, we will determine how and when patients decide to undergo surgery. Sixty nine percent of patients decided to undergo surgery prior to meeting the surgeon, while 47% stated that the surgeon had no influence on their decision. Before consulting with the surgeon, some patients gathered information from a variety of sources. Other healthcare professionals account for 81% of patients and family members 58%. Moreover, 68% of patients viewed iMed as a legal formality that had little impact on their decision. Hall et al. and his colleagues recommend that future research investigate whether patient decision regarding surgery is improved if healthcare practitioners introduce patients to educational resources such as iMed close to the time of initial diagnosis and prior to meeting the surgeon [15]. In contrast, only randomized clinical studies were included in the 2014 review by Gurusamy et al., comparing the advantages and disadvantages of formal patient education prior to laparoscopic cholecystectomy [16].

There are numerous methods for educating patients, such as PowerPoint presentations, DVD and computer-based multimedia programs, and verbal education. Due to the low quality of the existing evidence, the effects of formal education in addition to standard practice remain ambiguous, and well-designed trials are required [16]. As stated previously, informed consent should encompass all aspects of the condition, ensuring an adequate level of understanding and comprehension preoperatively, intraoperatively, and postoperatively, as well as the possibility of complications.

Mesri and his colleagues suggested that a more consistent and transparent consenting process is required, as evidenced by their 2021 study assessing the quality of the informed consent process for patients scheduled for conventional cholecystectomy but who instead undergo subtotal cholecystectomy [17]. Mesri and colleagues analyzed 174 consent forms between 2011 and 2017 and reported that only 5.2% of 174 patients had been apprised of the possibility of subtotal cholecystectomy [17]. Walming and his colleagues explored a novel educational technique as they investigated the impact of group consultation participation prior to colorectal cancer treatment and the results demonstrated that the majority of patients would recommend group consultation to others with the same condition; 81% agreed totally or partially that group counseling increases their sense of control; and 89% for active participation in their management, both of which have the potential to accelerate the recovery [18].

As stated previously, patient education and counseling should simplify and clarify the condition, surgery, postoperative care, and potential complications. Consequently, an evaluation of the most reliable method is required because it has a significant impact on the patient's knowledge base, which is required to make the final decision. This study aimed to determine the levels of surgical patients' education regarding surgical interventions among patients in Saudi Arabia. The specific objectives are to identify the function of surgeons in patient education, the most common information source used by patients, and the most common method of patient education.

## Materials and methods

Study design and population

This was an online survey study that was conducted between January and May 2023 to determine the levels of surgical patients' education regarding surgical interventions among patients in Saudi Arabia. The study population was patients who underwent surgeries (Elective, Emergency) living in Saudi Arabia. The inclusion criteria were patients who underwent surgeries or their caregivers if the patient was younger than 18 years and living in Saudi Arabia. Medically Free patients, younger than 18 years of age, not a citizen or resident of Saudi Arabia were excluded from this study.

Sampling procedure

Participants were recruited to participate in this study using a convenient sampling technique. Participants were invited through social media websites (Twitter, Telegram, WhatsApp, and Snapchat). In addition, data collectors around the kingdom were recruited to collect data and interview patients at different health care centers. Data collectors were trained to administer the questionnaire to the participants in a consistent manner to enhance consistency and standardization, minimize bias, and enhance their communication skills. Participants were given the option to continue participating in the study or withdraw on the first page of the questionnaire, which also included an informed consent form. The research's objectives were fully explained to patients to ensure that they understood the importance of their involvement. The inclusion criteria were listed in the letter of invitation for the study. Only patients who met the inclusion criteria were permitted to participate in the study. There was no compensation for their participation in the study to avoid potential bias.

Study tool

The questionnaire tool used in this study was developed based on a literature review in the field of patients' education regarding surgical interventions [4,19,20]. The questionnaire tool asked the participants about their demographic characteristics, previous surgery history profile, and characteristics of their communication with their treating surgeon. They were asked whether the treating surgeon (consultant surgeon or a member of his surgical team) explained the stages of reaching a diagnosis and the steps he will take to diagnose the patient's condition, whether he discussed the diagnosis with the patient, and how certain are they of the diagnosis, the medical staff who spoke about the diagnosis or access plan, whether the surgical procedure, method, and purpose been discussed with the patient before signing the consent document, whether the surgery described clearly and simply, whether alternatives other than surgical intervention offered, whether the patient has been informed of possible postoperative complications, and whether the entire postoperative treatment plan discussed. In addition, they were asked about the additional callout (a graphic or textual element used in various forms of visual communication to highlight a particular portion of an image, document, or presentation. Frequently, callouts are used to provide additional information and explanations regarding the highlighted content) used during the discussion by the medical provider, what callout was used in the discussion, whether what was discussed before the operation matched what was experienced after it, who spoke to the patient after the surgery and before he left the hospital, their assessment of the callouts and the method used by the doctor, the extent to which they needed an external source to understand the diagnosis, surgery, possible complications, as well as the treatment plan and follow-up in general, what are the external sources that were resorted to, and how do they evaluate the quality of communication by the consultant surgeon himself in general.

Piloting phase and survey testing

The questionnaire instrument was evaluated and confirmed by medical professionals from the College of Medicine at Imam Mohammad Ibn Saud Islamic University, Riyadh, Saudi Arabia. The questionnaire was evaluated in terms of the clarity of the questions, suitability of the language, and overall structure of the survey. This included question clarity and wording, response options, survey flow, survey length, and survey formatting. Participants were questioned on the questions' clarity, comprehensibility, and face validity as well as whether any of them were challenging to comprehend. They claimed that it was simple to comprehend and finish the questionnaire. Additionally, before the questionnaire is widely used, a pilot study was conducted with a small sample of the study population to assess their understanding of the questionnaire. The pilot study involved 30 participants from our target population. They completed the questionnaire of the study and confirmed that the questions are clear and met the study objectives.

Sample size

Using a 95% confidence interval, a 0.5 standard deviation (SD), and a 5% margin of error, the minimum required sample size was 385 individuals.

Ethical approval

Imam Mohammad Ibn Saud Islamic University's Institutional Review Board in Riyadh, Saudi Arabia gave its approval for this study (360/2022). The research ethics committee gave its approval to the waiver of consent because participation in the study was voluntary.

Statistical analysis

The data from this study were analyzed with IBM SPSS Statistics for Windows, Version 27 (Released 2020; IBM Corp., Armonk, New York, United States). In order to present categorical variables, frequency and percentage were used. To find indicators of satisfaction with the callouts and the surgeon-consultant's communication quality, binary logistic regression analysis was utilized. Statistical significance was determined using a two-sided p-value of less than 0.05.

## Results

Baseline characteristics of the study participants

A total of 1360 participants were involved in this study. More than half of them (61.4%) were females. Almost 44.0% of them were aged 18-30 years. The vast majority of them (93.7%) were Saudis. Almost one-third of them (34.9%) were living in the Southern area. More than half of them (67.6%) reported that they hold a bachelor's degree.

Around one-third of the study participants (36.5%) reported that either themselves or a family member or friend underwent a surgical procedure that require general/local anesthesia in the past, and they were aware of the details of the condition while they were with him/her in the hospital for emergency operations. Almost one-third (33.2%) of the operations were performed after 2020. Around 41.0% of the operation were performed in Riyadh city. For further details of the baseline characteristics of the study participants, refer to Table 1.

**Table 1:** Baseline characteristics of the study participants

Variable	Frequency	Percentage
Gender		
Females	835	61.4%
Age category		
18-30 years	593	43.6%
31-40 years	314	23.1%
41-50 years	260	19.1%
51-60 years	136	10.0%
61 years and above	57	4.2%
Nationality		
Saudi	1274	93.7%
Area of residency		
Eastern area	192	14.1%
Western area	301	22.1%
Northern area	56	4.1%
Southern area	475	34.9%
Central area	336	24.7%
Education level		
High school level or lower	335	24.6%
Bachelor level	919	67.6%
Higher education	106	7.8%
Please specify the type of surgery (emergency or non-emergency) that you or a family member or friend underwent in the past, and you were aware of the details of his condition while you were with him in the hospital? (Non-emergency operation is a scheduled one that is scheduled in advance. Emergency cases are confirmed during the emergency department review)		
Emergency operation	496	36.5%
When was the operation?		
Before 2000	60	4.4%
2000-2005	76	5.6%
2006-2010	116	8.5%
2011-2015	175	12.9%
2016-2020	358	26.3%
After 2020	452	33.2%
I cannot determine the period	123	9.0%
In which area was the surgery performed?		
Riyadh	583	41.4%
Aseer	296	21.8%
Makkah	281	20.7%
Najran	78	5.7%
AlMadinah	52	3.8%
Hail	30	2.2%
AlQassim	19	1.4%
Jazan	15	1.1%
Tabuk	9	0.7%
AlBahah	10	0.7%
Northern area	4	0.3%
AlJowf	3	0.2%
The hospital where the surgery took place:		
Governmental hospital (affiliated to the Ministry of Health)	655	48.2%
Private hospital	474	34.9%
Governmental hospital (not affiliated with the Ministry of Health as hospitals military sectors)	231	17.0%

Patients' education profile

Table 2 presents the patients' education profile of the study participants. When the participants were asked “When was their first meeting with the consultant surgeon who performed the operation and was responsible for your condition”, around 40.5% of them reported that they met him after diagnosing the condition and referring it to him. Around 70.0% of the study participants reported that the consultant surgeon himself spoke to them about a diagnosis or a plan to reach it and discussed the surgical procedure, method, and purpose with them before signing the consent document.

**Table 2:** Patients' education profile

Variable	Frequency	Percentage
When was your first meeting with the consultant surgeon who performed the operation and was responsible for your condition?		
I met him after diagnosing the condition and referring it to him	551	40.5%
I met him before I knew about the diagnosis	428	31.5%
I met him for the first time before the operation in the operating room	164	12.1%
I'm not sure if I ever met him, I didn't recognize him	110	8.1%
I met him for the first time after the operation, before discharge from the hospital	82	6.0%
I met him for the first time after the operation at the follow-up visit after	25	1.8%
Has your doctor (consulting surgeon or a member of his surgical team) explained the stages of reaching a diagnosis to you and the steps he/she will take to diagnose your condition? (Example: the need for additional clinical examinations, blood samples, or x-rays)		
Yes, I was already aware of the steps to reach the diagnosis	1131	83.2%
No, I was not aware in advance, I do not know how they arrived at the diagnosis	229	16.8%
Did he/she or someone on his/her staff discuss the diagnosis with you and how certain are they of the diagnosis? (Based on what was reached after taking the medical history and clinical, laboratory, and medical imaging examination)		
Yes	1198	88.1%
Who spoke to you about a diagnosis, or a plan to reach it? (More than one option can be selected)		
The consultant surgeon himself	953	70.1%
A member of the surgical team	486	35.7%
Another doctor who is not on the surgical team	156	11.5%
The nursing staff	83	6.1%
Other	74	5.4%
Has the surgical procedure, method and purpose been discussed with you before signing the consent document? (More than one option can be selected)		
Yes, the consultant surgeon himself	978	71.9%
A member of the surgical team	450	33.1%
The nursing staff	99	7.3%
Another doctor who is not on the surgical team	79	5.8%
Other	48	3.5%
No, there was no discussion, I just signed	77	5.7%
Was the surgery described clearly and simply? (As the steps of the process - the techniques used)		
Yes	1163	85.5%
Are alternatives other than surgical intervention offered? (In cases where there is an alternative to the surgical solution)		
Yes	467	63.1%
Have you been informed of possible postoperative complications?		
Yes	1082	79.6%
Was the entire postoperative treatment plan discussed?) This includes the period of admission - post-operative personal care - post-operative instructions - pain management methods (		
Yes	1113	81.8%
Was an additional callout used during the discussion by the medical provider? (The consultant surgeon himself - the emergency physician - the anesthesiologist - the nursing staff)		
Yes	1106	81.3%
From your point of view, did what was discussed with you before the operation match what you experienced yourself after it?		
Yes	946	69.6%
No	104	7.6%
Partial match	310	22.8%
Were there complications from the surgery that were not discussed with you or were you aware of the possibility of them occurring before you entered the surgery?		
Yes	336	24.7%
Was the rest of the treatment plan after the operation and the follow-up plan discussed before discharge from the hospital? (Such as following a specific diet, surgical wound care, and personal hygiene)		
Yes	1150	84.6%
Who spoke to you after the surgery and before you left the hospital? (More than one option can be selected)		
The consultant surgeon himself	941	69.2%
A member of the surgical team	509	37.4%
The nursing staff	187	13.8%
Another doctor who is not on the surgical team	114	8.4%
Other	49	3.6%
What is your assessment of the callouts and the method used by your doctor?) Was it sufficient to know all aspects of the case before the surgical intervention, starting from the diagnosis of the case and ending with the follow-up plan before discharge from the hospital? (		
Excellent	576	42.4%
Very good	399	29.3%
Good	326	24.0%
Bad	40	2.9%
Very bad	19	1.4%
How do you assess the extent to which you need an external source to understand the diagnosis, surgery, possible complications, as well as the treatment plan and follow-up in general after the discussion that your doctor had with you - if it happened?		
Yes, a total need	419	30.8%
Yes, average need	448	32.9%
There was no need at all	493	36.3%
How do you evaluate the quality of communication by the surgeon-consultant himself with you or with the companions in general?		
Excellent	981	72.1%
Good	318	23.4%
Bad	40	2.9%
Very bad	21	1.5%

The vast majority of the study participants reported that the consulting surgeon or a member of his surgical team explained the stages of reaching a diagnosis for them and the steps he/she will take to diagnose their condition (83.2%), discussed the diagnosis with them and how certain are they of the diagnosis (88.1%), described the surgery clearly and simply (85.5%), informed them of alternatives other than surgical intervention (63.1%), discussed the entire postoperative treatment plan (81.8%), informed of possible postoperative complications (79.6%), used additional callout during the discussion (81.3%), and spoke to them after the surgery and before they left the hospital (69.2%).

The majority of the participants (70.0%) reported that what was discussed with them before the operation matched what they experienced after it. The vast majority of the study participants (95.7%) reported that the callouts and the method used by their doctor were sufficient to know all aspects of the case before the surgical intervention, starting from the diagnosis of the case and ending with the follow-up plan before discharge from the hospital. Similarly, 95.5% of the study participants reported that they were satisfied with the quality of communication by the surgeon-consultant himself with them or with the companions in general.

Around one-third of the study participants (36.3%) reported that they did not need an external source to understand the diagnosis, surgery, possible complications, and the treatment plan and follow-up in general after the discussion that your doctor had with them.

**Figure 1:**
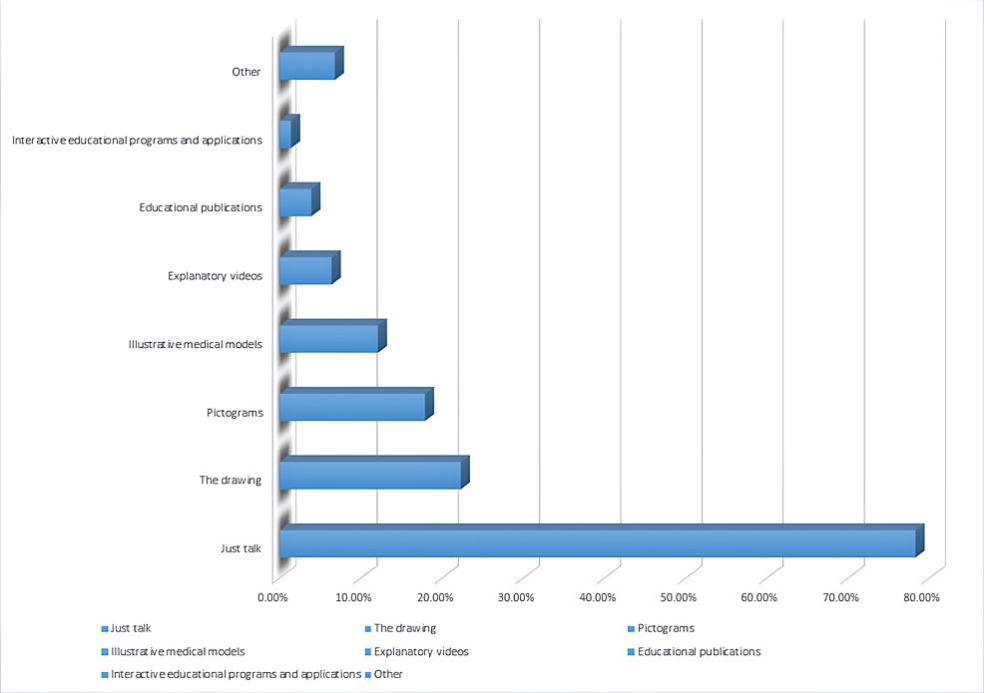
Callout used in patients' discussion

The most commonly used callouts during patients’ education were discussion “Just talk”, drawing, and pictograms, accounting for 78.3%, 22.3%, and 17.9%, respectively, Figure 1.

**Figure 2:**
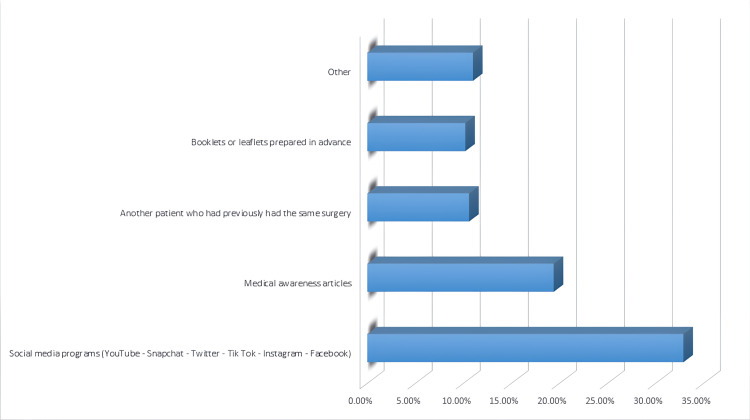
The external sources that have been used by the study participants

For participants who reported that they needed to access external sources to understand the diagnosis, surgery, possible complications, and the treatment plan and follow-up in general, the most commonly reported source was social media programs (YouTube, Snapchat, Twitter, TikTok, Instagram, and Facebook) (32.9%), Figure 2.

Predictors of being satisfied with educational services provided by surgeons.

Binary logistic regression analysis identified that there is no statistically significant difference in participants’ satisfaction with the callouts and the method provided by surgeons based on their baseline demographic characteristics. However, Saudi participants were more likely to be satisfied with the quality of communication by the surgeon-consultant (p<0.05). At the same time, participants who live in the Southern area were less likely to be satisfied with the quality of communication by the surgeon-consultant (p<0.05), Table 3.

**Table 3:** Predictors of being satisfied with educational services provided by surgeons *p<0.05

Variable	Satisfaction with the callouts and the method used (95% confidence interval)	Satisfaction with the quality of communication by the surgeon-consultant (95% confidence interval)
Gender		
Females (Reference group)	1.00	
Males	0.82 (0.43-1.58)	1.47 (0.88-2.46)
Age category		
18-30 years (Reference group)	1.00	
31-40 years	0.57 (0.29-1.10)	0.60 (0.35-1.04)
41-50 years	0.97 (0.45-2.14)	0.78 (0.43-1.45)
51-60 years	1.03 (0.36-2.93)	0.85 (0.38-.91)
61 years and above	-	0.84 (0.26-2.76)
Nationality		
Non-Saudi (Reference group)	1.00	
Saudi	0.75 (0.18-3.18)	2.36 (1.09-5.14) *
Area of residency		
Eastern area (Reference group)	1.00	
Western area	1.18 (0.54-2.58)	1.47 (0.74-2.94)
Northern area	0.53 (0.16-1.77)	0.37 (0.15-0.89) *
Southern area	1.31 (0.66-2.59)	1.20 (0.69-2.08)
Central area	0.70 (0.36-1.37)	0.71 (0.41-1.25)
Education level		
High school level or lower (Reference group)	1.00	
Bachelor level	0.76 (0.38-1.53)	0.87 (0.49-1.52)
Higher education	0.60 (0.23-1.56)	0.64 (0.28-1.44)
Please specify the type of surgery (emergency or non-emergency) that you or a family member or friend underwent in the past, and you were aware of the details of his condition while you were with him in the hospital?		
Non-emergency operation (Reference group)	1.00	
Emergency operation	1.24 (0.64-2.43)	0.88 (0.52-1.49)
When was the operation?		
Before 2000 (Reference group)	1.00	
2000-2005	0.41 (0.16-1.08)	0.84 (0.30-2.37)
2006-2010	3.82 (0.52-28.05)	1.84 (0.57-5.97)
2011-2015	0.71 (0.31-1.63)	0.59 (0.31-1.13)
2016-2020	0.68 (0.35-1.31)	0.85 (0.48-1.49)
After 2020	2.09 (0.96-4.57)	1.02 (0.59-1.77)
The hospital where the surgery took place:		
Governmental hospital (affiliated to the Ministry of Health) (Reference group)	1.00	
Private hospital	1.20 (0.50-2.89)	2.36 (0.93-5.96)
Governmental hospital (not affiliated with the Ministry of Health as hospitals military sectors)	1.03 (0.54-1.99)	1.01 (0.64-1.90)

## Discussion

This study aims to evaluate community education regarding the procedure, potential complications, risks, and knowledge that can be used to evaluate the efficacy of health care providers' communication and identify patients' needs.

Around 40.5% of study participants reported that their first encounter with the consultant surgeon who performed the operation and was responsible for their condition occurred after the condition was diagnosed and referred to him. Indeed, this is the ideal process where the surgeon or the assigned nurse meets the patient and provides the necessary information prior to surgery, including appointment information, required pre-admission tests, and surgery procedures and instructions [21], while approximately 70% of the study participants reported that the consultant surgeon himself had discussed the surgical procedure, method, and purpose with them prior to the surgery. While the presence of the consultant surgeon is essential, the role of nurses in this preoperative process has become increasingly important in reducing adverse patient outcomes. Physicians are asked by an ARON guidance statement to allow perioperative nurses to engage more with patients and increase their contact with them in a way that improves patient safety, care, treatment, and pain management, resulting in a decreased risk for adverse patient outcomes [1,22].

The vast majority of the study participants (83.2%) reported that the consulting surgeon or a member of his surgical team explained the stages of reaching a diagnosis for them and the steps he/she will take to diagnose their condition, where educating patients and explaining the diagnosis with the patients by healthcare providers is highly important and considered a large part of standard care [23]. A total of 88.1% of the study participants reported that the consulting surgeon or a member of his surgical team discussed the diagnosis with them and how certain are they of the diagnosis, where this patient case review may offer the chance to also understand and assess patient knowledge and behavior toward the diagnosis, and this may be used to connect patients old knowledge with the precise, corrected, and patient case newer one in order to promote patient understanding of their cases [24]. This is a good clinical practice indicator where the patient's understanding often is presumed and scarcely officially assessed [20]. Also 85.5% of the study participants reported that the consulting surgeon or a member of his surgical team described the surgery clearly and simply, which is consistent with the North American practice of respecting patient literacy, using simple language, and avoiding the use of difficult medical terminology in order to facilitate and ensure a better understanding of the surgery [24,25]. However, 63.1% of the study participants reported that the consulting surgeon or a member of his surgical team discussed alternatives to surgical intervention, 81.8% discussed the entire postoperative treatment plan, and 79.0% informed of possible postoperative complications; in fact, introducing all treatment pathways and their alternatives is essential, where the treatment choice is based on the patient's preference with guidance. In addition, 81.3% of the study participants reported that the consulting surgeon or a member of his surgical team used additional callouts during the discussion, and 69.2% spoke with them after the surgery and prior to their discharge from the hospital. Using additional callouts during the discussion with patients is key to improving the patient's understanding of the procedures and instructions for the operation process [24,26], and having a discussion with the patient after surgery and prior to discharge aids in assessing patient needs, reducing patient anxiety, and promoting patient self-care [27].

The majority of participants (70%) reported that what was discussed with them prior to the procedure corresponded to what they personally experienced afterward. 95.7% of study participants reported that the callouts and methods used by their physician were sufficient to know all aspects of the case prior to surgical intervention, beginning with the diagnosis and concluding with the follow-up plan prior to hospital discharge. This substantially demonstrates the effect of preoperative patient education received and its value in the preoperative, intraoperative, and postoperative phases, where it plays a major role in patients' information and comprehension, as well as psychological and social support [28]. Similarly, 95.5% of the study participants reported that they were satisfied with the quality of communication by the surgeon-consultant himself with them or with their companions in general. This is consistent with research that generally demonstrates surgeons are great at providing information about surgical conditions and considering the surgical process and details [29,30]. Surgeons play an important and efficient role in informed decision-making during surgery.

About one-third of study participants (36.3%) reported that they did not need an external source to understand the diagnosis, surgery, possible complications, treatment plan, and follow-up in general after the doctor's discussion with them, where the need for external callout depends on patients' needs and the assessment of patients' understanding and knowledge by health care providers [21,22]. Indeed, discussion "Just talk", drawing, and pictograms were the most frequently used callouts during patients' education, accounting for 78.3%, 22.0%, and 17.9%, respectively, and this is the common explanation pathway used during discussion of any health care provider with the patient [21,24,31]. Verbal education is an essential call-out tool for patient education, especially when using simple language during the discussion with patients who have low health literacy. The most commonly reported external source was social media programs (YouTube, Snapchat, Twitter, Tik Tok, Instagram, and Facebook), which accounted for 32.9% of participants who needed external sources to understand the diagnosis, surgery, possible complications, treatment plan, and follow-up in general. The use of multimedia education is known to significantly increase and improve patient understanding [6-9].

Saudi participants were more likely to be satisfied with the quality of communication by the surgeon-consultant, and this satisfaction is related to the great communication between the surgeon and the patients, as surgeons are known to be exceptional in discussing surgical conditions, details, and approaches [29]. Indeed, positive physicians' behavior and patients' partnership building is significantly affecting the quality of physician-patient communication; therefore, Saudi participants were more likely to be satisfied with the quality of communication by the surgeon-consultant. Simultaneously, participants living in the South were less likely to be satisfied with the quality of communication by the surgeon-consultant, and this dissatisfaction is associated with the lack of surgeon attention to non-medical patient's issues [29,32,33], where they fail to understand patients' emotions or concerns and also fail to demonstrate empathy [34]. Also, other factors may play a significant role in this dissatisfaction, such as clarity of the patient about their doubts and complaints, where patients who do not mention or discuss their worries and concerns, which is 53% of the time [35], feel that there is a gap that contributes to their dissatisfaction [36], as well as the increased workload and pressure on surgeons, which makes it difficult to give enough time to the education process; therefore, patients are less likely to be satisfied.

In fact, the surgeon's role in preoperative patient education is essential, and the surgical team's involvement in this process is substantial; consequently, certain guidelines and roles must be established to maintain and improve the quality of surgeon-patient communication, which affects preoperative, operative, and postoperative patient knowledge and understanding, as well as assisting in the selection of treatment approaches and the provision of informed consent. Also recommended is the use of multiple callouts to improve patients' comprehension of their cases and treatment options.

Study limitations

This research has limitations. It is possible that a substantial portion of the target population does not have access to social media websites; therefore, the use of an online survey to recruit study participants is subject to criticism. According to the most recent data from 2023, approximately 79.3% of the Saudi Arabian population uses social media. The cross-sectional study design constrained our ability to observe causal relationships between study variables. Due to the use of convenience sampling, the generalizability of our study's findings may be limited. It is important to keep in mind that participants' responses in our study are not necessarily based on a recent recall of the events being reported and based on the research of long-term memory, which might have affected the validity of the findings.

## Conclusions

The study emphasizes the critical role of surgeons in preoperative patient education and the significance of surgical team participation in this process. It is necessary to establish guidelines and roles to strengthen surgeon-patient communication in order to increase patient knowledge, facilitate treatment decisions, ensure informed consent, increase patient and surgeon awareness, foster patient concern expression, and promote non-medical patient engagement.

## Appendices

Questionnaire instrument

Demographic Characteristics Section

**Table 4:** Demographic characteristics section

Variable
Gender
Males
Females
Age category
18-30 years
31-40 years
41-50 years
51-60 years
61 years and above
Nationality
Saudi
Non-Saudi
Area of residency
Eastern area
Western area
Northern area
Southern area
Central area
Education level
High school level or lower
Bachelor level
Higher education
Please specify the type of surgery (emergency or non-emergency) that you or a family member or friend underwent in the past, and you were aware of the details of his condition while you were with him in the hospital? (Non-emergency operation is a scheduled one that are scheduled in advance. Emergency cases are confirmed during the emergency department review)
Emergency operation
Non-emergency operation
When was the operation?
Before 2000
2000-2005
2006-2010
2011-2015
2016-2020
After 2020
I cannot determine the period
In which area was the surgery performed?
Riyadh
Aseer
Makkah
Najran
AlMadinah
Hail
AlQassim
Jazan
Tabuk
AlBahah
Northern area
AlJowf
The hospital where the surgery took place:
Governmental hospital (affiliated to the Ministry of Health)
Private hospital
Governmental hospital (not affiliated with the Ministry of Health as hospitals military sectors)

Patients' Education Profile

**Table 5:** Patients education profile

Variable
When was your first meeting with the consultant surgeon who performed the operation and was responsible for your condition?
I met him after diagnosing the condition and referring it to him
I met him before I knew about the diagnosis
I met him for the first time before the operation in the operating room
I'm not sure if I ever met him, I didn't recognize him
I met him for the first time after the operation, before discharge from the hospital
I met him for the first time after the operation at the follow-up visit after
Has your doctor (consulting surgeon or a member of his surgical team) explained the stages of reaching a diagnosis to you and the steps he/she will take to diagnose your condition? (Example: the need for additional clinical examinations, blood samples, or x-rays)
Yes, I was already aware of the steps to reach the diagnosis
No, I was not aware in advance, I do not know how they arrived at the diagnosis
Did he/she or someone on his/her staff discuss the diagnosis with you and how certain are they of the diagnosis? (Based on what was reached after taking the medical history and clinical, laboratory and medical imaging examination)
No
Yes
Who spoke to you about a diagnosis, or a plan reach it? (More than one option can be selected)
The consultant surgeon himself
A member of the surgical team
Another doctor who is not on the surgical team
The nursing staff
Other
Has the surgical procedure, method and purpose been discussed with you before signing the consent document? (More than one option can be selected)
Yes, the consultant surgeon himself
A member of the surgical team
The nursing staff
Another doctor who is not on the surgical team
Other
No, there was no discussion, I just signed
Was the surgery described clearly and simply? (As the steps of the process - the techniques used)
No
Yes
Are alternatives other than surgical intervention offered? (In cases where there is an alternative to the surgical solution)
No
Yes
Have you been informed of possible postoperative complications?
No
Yes
Was the entire postoperative treatment plan discussed?) This includes the period of admission - post-operative personal care - post-operative instructions - pain management methods (
No
Yes
Was an additional callout used during the discussion by the medical provider? (The consultant surgeon himself - the emergency physician - the anesthesiologist - the nursing staff)
No
Yes
From your point of view, did what was discussed with you before the operation match what you experienced yourself after it?
Yes
No
Partial match
Were there complications from the surgery that were not discussed with you or were you aware of the possibility of them occurring before you entered the surgery?
No
Yes
Was the rest of the treatment plan after the operation and the follow-up plan discussed before discharge from the hospital? (Such as following a specific diet, surgical wound care, and personal hygiene)
No
Yes
Who spoke to you after the surgery and before you left the hospital? (More than one option can be selected)
The consultant surgeon himself
A member of the surgical team
The nursing staff
Another doctor who is not on the surgical team
Other
What is your assessment of the callouts and the method used by your doctor?) Was it sufficient to know all aspects of the case before the surgical intervention, starting from the diagnosis of the case and ending with the follow-up plan before discharge from the hospital? (
Excellent
Very good
Good
Bad
Very bad
How do you assess the extent to which you need an external source to understand the diagnosis, surgery, possible complications, as well as the treatment plan and follow-up in general after the discussion that your doctor had with you - if it happened?
Yes, a total need
Yes, average need
There was no need at all
How do you evaluate the quality of communication by the surgeon-consultant himself with you or with the companions in general?
Excellent
Good
Bad
Very bad

**Table 6:** Callouts and external sources

Variable
Used callout during patients’ education:
Interactive educational programs and applications
Educational publications
Explanatory videos
Illustrative medical models
Pictograms
The drawing
Just talk
External sources needed to understand the diagnosis, surgery, possible complications, as well as the treatment plan and follow-up in general:
Booklets or leaflets prepared in advance
Another patient who had previously had the same surgery
Medical awarenss articles
Social media programs (YouTube, SnapChat, Twitter, TikTok, Instagram, Facebbok)
Others
